# Remote Teaching in a Rwandan Emergency Medicine Residency: A Viable Option with Limited In-person Staff

**DOI:** 10.24248/eahrj.v8i3.801

**Published:** 2025-01-30

**Authors:** Andrew Beck, Maria Isabel Diaz, Enyonam Odoom, Claudien Niyigirimbabazi, Oriane Longerstaey, Vincent Ndebwanimana, Doris Uwamahoro, Naz Karim

**Affiliations:** a Brown University, Department of Emergency Medicine, 55 Claverick St, Suite 100, Providence, RI, 02903, USA; b University Teaching Hospital of Kigali (CHUK), KN 4 Ave, Kigali, Rwanda.

## Abstract

Low and middle-income countries (LMIC) in Africa face challenges in medical education. Eleven countries have no medical school, 24 countries share one medical school, and few have residency programs. This shortage means that Africa has only 1.7% of the world’s physicians, yet bears 27% of the global disease burden. COVID-19 created further educational constraints, especially in emergency medicine (EM). Student and resident education opportunities were limited. Rwanda faced a shortage of available in-person EM residency instructors during the pandemic, and to support learning needs, we designed and implemented a remote teaching model to substitute in-person instruction. The objective of this study was to evaluate whether remote and pre-recorded teaching is positively received by EM learners and if it is a viable supplement in resource limited settings.

Pre-recorded lectures were presented to residents, with lecturers remotely available. We evaluated the program using the first-level Kirkpatrick framework (suitability/satisfaction) via a quantitative and qualitative post-lecture survey. The survey was completed by residents in attendance. Responses were analyzed using descriptive statistics. Outcome measures included learner satisfaction, lecture quality, technological quality, and situational suitability. Qualitative and free-response data was An average of 11 Rwandan EM residents attended 18 lectures. Using a Likert scale, the composite learner satisfaction score was 4.25 (σ = 0.1), the lecture quality score was 4.2 (σ = 0.1), the technological quality score was 4.0 (σ = 0.36), and the situational suitability score was 4.25 (σ = 0.07). These results indicated overall satisfaction with the lectures. Lower scores were given regarding lecturer accents and speech rates. Qualitative feedback did not demonstrate significant dissatisfaction with quality or suitability.

When in-person lecturers are unavailable, pre-recorded, remote instructional methods may be an appropriate substitute. Future directions may include piloting the project with a larger, multinational cohort or in LMICs with greater technological or resource limitations.

## BACKGROUND

Low and middle-income countries (LMICs) in Africa face challenges in undergraduate and graduate medical education. Such challenges include a shortage of medical schools: only one medical school serves 24 countries in Africa, while 11 countries have no medical school at all. Advances have been made in the last 10 years, however, there remains work to be done, as many medical schools or residency programs are nascent and have yet to deliver adequate supply of trained, accredited physicians.^1–3^ A lack of residencies compounds the challenge, and the establishment of formalized residency programs throughout LMICs in Africa remains nascent. This is especially true in emergency medicine (EM).^4–6^ Only 15 formal EM residencies existed in Africa as of 2017.^5–6^ Heterogeneity also exists across the residencies.^7^ The cascading effect of limitations in the number of physicians and trained specialists is far-reaching. Africa has 1.7% of the world’s physicians while bearing 27% of the global burden of disease.^8^ By contrast, in high-income countries (HIC), ample schools and residency programs exist, and technological advances allow for both traditional in-person learning plus blended and asynchronous learning.^9–13^ Such models involve several interactive methodologies: formal didactic and in-person lectures, textbook or journal reading, asynchronous and online learning exercises, pre-recorded and remote lectures, in-person case simulations, oral and written quizzes and examinations, bedside teaching, question banks, and peer teaching.^14^ Blended models provide multimodal exposure to topics for stimulation of engagement and retention in adult learners while also permitting flexibility when in-person instructors are not available.^15^ Such breadth of teaching comes at a financial and operational cost to instructors and institutions. Such cost limits the viability of an identical or multimodal educational model in LMICs.^16^ Additionally, LMICs have significant financial and operational limitations that render the implementation of e-learning methods difficult and make learning gaps more pronounced.^17–20^ These effects were especially striking during the COVID-19 pandemic, due to both staff shortages or shifting resource demands and the need to adapt educational models to prevent in-person congregation. Many institutions within both HICs and LMICs had challenges adjusting their learning models to permit remote or asynchronous learning during this time, resulting in educational gaps compounded by limited availability of instructors.^21,22^

Like most African countries, Rwanda faced similar challenges.^15,23–25^ Many EM providers were financially unable to continue working in academic facilities thus limiting the number of instructors able to teach residents about emergency care. The shortage of qualified instructors and educational staff necessitated the initiation of supplemental learning models.^15,23^ While the use of asynchronous, online, and remote learning models is common in HICs, the delivery of educational content via these mechanisms in LMICs is less common, and less studied.^26^ It has been used outside of the EM residency setting within Rwandan healthcare education with adequate learner satisfaction, though challenges exist including alignment of content to need, technological adequacy, and usability.^27,28^ Recognizing the potential of remote learning to assist the Rwandan EM residency at the University Teaching Hospital of Kigali (CHUK) during pandemic teaching constraints, we pre-recorded and delivered 18 video lectures in EM education. This work was developed at the request of the EM residency director, with the goal of substituting in-person education with remote and online teaching. Each lecture was delivered, with the lecturer available remotely via Zoom for questions. Such curricular delivery is novel in LMICs and may constitute an evidence-based innovation that could transform the learning environment in LMICs where healthcare workforce constraints continue to limit learning opportunities. The objective of this research is to evaluate how emergency remote teaching (in the form of pre-recorded lectures) is perceived by EM resident learners, and to consider whether it is a viable supplement when an in-person instructor is not available to teach in an LMIC.

## METHODS

### Type and Period of Study

The study design was a prospective, non-randomized, learner satisfaction study involving Rwandan EM residents. The study period took place from 2021–2022 followed by data collection and analysis.

### Study Population

Participants in this study represent the census of CHUK EM residents. EM medical students and allied-health staff from the CHUK Faculty of Medicine were allowed to participate in the lectures but did not complete evaluations. An average of 11 Rwandan EM residents [range: 5–17] participated per session. The group of surveyed and analyzed participants included the residents within the CHUK EM residency. There were no exclusion criteria for EM resident participation, as participation in didactic learning is mandatory within the residency. The only inclusion criterion was status as a resident within the CHUK EM residency.

### Content Creation

Eighteen lectures were pre-recorded on a voluntary, non-compensated basis by USA-based academic Global Emergency Medicine faculty with qualifications at or above the clinical instructor level. Required lecturer qualifications included at least one-to-five years of experience in global emergency medicine teaching and research, and academic appointments. Lectures were recorded using Zoom ® (Zoom Video Communications, San Jose, CA), and were reviewed to assure correct teaching along with error-free video and audio. Files were then stored on a cloud platform, Google ® drive (Google/Alphabet, Inc., Mountain View, CA) and downloaded to local computers in Rwanda. Lectures ranged from 30 minutes to 1.5 hours in duration and covered topics considered fundamental to EM care. Topics were chosen by EM faculty within the Rwandan EM residency based on areas of greatest learning need and were reviewed by the EM residency director for appropriateness and suitability. Theoretical and practical aspects of EM were taught in each video, with foundational knowledge followed by clinical applicability. This study focused on the development and delivery of EM content only due to the specific request for this from CHUK EM faculty.

### Lecture Delivery

Two pre-recorded lectures per session were played in a CHUK classroom via audiovisual projector after downloading from the cloud storage location. A research assistant (EO) was responsible for downloading, setting up, confirming audio and video quality, troubleshooting, and playing content for attendees. Throughout the lecture, US-based instructors were available via Zoom to pause playback and answer questions in real time. Participants received access to recordings and slides for review from personal computers. Computers, speakers, projectors, and screens were PC-based; quality varied based on location availability within CHUK on days of lectures.

### Data Collection and Analysis

Program evaluation was designed around the first-level Kirkpatrick framework. A post-lecture questionnaire was administered by an in-person research assistant (EO) to each EM resident participant (only the EM residents were evaluated) after each session (Table 2). Questionnaires addressed outcome measures of learner satisfaction, lecture quality, technological quality, and situational suitability using a Likert scale. Responses were anonymous. The only identifying information collected was learner educational level (i.e., level within residency). Likert scale responses were analyzed using Microsoft Excel to compute means and standard deviations. Free responses were analyzed using qualitative analysis. Questionnaires were translated into Kinyarwanda and free responses were translated into English.

### Ethical Considerations

The Institutional Review Board and Ethics Committee of Brown University determined the study was of minimal risk to participants (#1747880, 007121). Before participating, participants were informed about the research aims and were given the opportunity to ask questions related to their involvement. Information about voluntary participation, right to withdraw from participation, and any needed clarification was provided to the participants via the informed consent process. Every participant signed a written informed consent form before participation in this research. Regarding confidentiality, the collected data were not used for purposes other than those mentioned in the objectives of this study, and personal identifying information that could compromise the educational process was not collected.

## RESULTS

An average of 11 Rwandan EM residents [range: 5–17] attended 18 lectures. From this group, 219 questionnaires were administered (one questionnaire/participant/session). Residency year was reported (Table 1).

**Table 1:** Resident Participants by Year

Year of Residency	Percentage of Participants
First Year	40.6%
Second Year	46.1%
Third Year	0.4%
Fourth Year	11%

**Table 2:** Descriptive Statistics of Likert Scale Survey Questions, and (outcome measure assessed): Qualitative Results

Questionnaire Item and (Outcome Measure)	Mean (out of 5) ± SD
1. Did the video-based lectures meet your expectations? (Learner Satisfaction, Lecture Quality, Technological Quality)	4.1 ± 1.1
2. Were the lectures free of technological errors? (Technological Quality)	3.6 ± 1.6
3. Would you recommend this method of learning to a peer? (Learner Satisfaction, Lecture Quality, Technological Quality, Situational Suitability)	4.3 ± 1.2
4. Was the length of each lecture appropriate? (Lecture Quality, Situational Suitability)	4.2 ± 1.2
5. Did the accompanying slides help you better understand the course material presented in the lectures? (Learner Satisfaction, Lecture Quality)	4.3 ± 1.0
6. Was the content relevant to your practice environment? (Learner Satisfaction, Situational Suitability)	4.3 ± 1.1
7. Did you have questions about the lectures that you wanted to ask, but were unable to ask because of technical difficulties? (Technological Quality)	1.1 ± 0.4
8. Did you have difficulty understanding the lecture due to the instructor's accents? (Lecture Quality, Situational Suitability)	2.0 ± 1.3
9. How effective were the instructors in teaching the content you learned today? (Lecture Quality)	4.1 ± 1.0

### Quantitative Results

Respondents completed questionnaires (Table 2) using a 5-point Likert scale. A response of “No” was coded as 1, “Somewhat” as 3, and “Yes” as 5. Averages and standard deviations were computed. Table 2 summarises these averages, with averages of 4.0 and higher indicating positive attitudes toward the e-learning modality. Learners reported that the lectures were of adequate quality, relevant to their curriculum, of appropriate length, effective, and recommendable to a peer. Based on the results, the primary area of improvement was the technological quality of the lectures.

### Qualitative Results

We performed a qualitative analysis of open-ended questions to gather participant perceptions on the lectures. Areas assessed included whether participants would recommend the learning method to peers, benefits of this teaching method, clinical relevance, organization and structure, and areas for improvement.

Regarding recommending the learning method: A majority said the teachings were helpful. Specifically, ‘teachings aided in interpretation of medical imaging technologies such as CT scans and gave residents the ability to compare EM care in Rwanda to other settings.’ Regarding benefits of this teaching method: Residents reported that teaching methods helped them meet required clinical competencies and improved their theoretical knowledge. One resident stated, ‘Good teaching method [sic] really encouraging.’

Regarding clinical relevance: Some residents expressed that the course helped in learning about patient management and that the information was supplementary to their program’s curriculum. One resident said, ‘This is very helpful because we get different explanations with different lecturers, this helps us to learn different knowledge.’

Regarding organization and structure: The perception of course organization and structure were positive; several learners reported that the lectures were well-detailed and had brief, precise explanations. Others expressed the value of having access to different facilitators from other parts of the world. One resident said, ‘It is the best method to learn from experts all over the world.’ Most residents said the teaching method was interesting, others stated the materials were understandable and well-explained.

Regarding areas for improvement, suggestions to improve future teaching included: Increased regularity of lectures, resident access to lecture slides and videos on the day of lecture, increased practical experience, a breakup of lengthy lectures, and additional clinical cases. Several participants complained of language barriers, which included complaints of quick speech and inability to pause lectures. One participant stated, ‘We may miss important context due to language barriers. Some residents reported inadequate audio quality, and electricity interruptions. One resident stated ‘We had stopped the presentation for at least 15 minutes because of heavy rain. We could not hear the presentation.’ Some residents said virtual teaching was boring, and others reported long and busy slides. Two residents reported their preference of in-person teaching, and others reported loss of concentration and limited interactions with lecturers. Some report the need for additional clinical practice and requested a summary of topics covered during the study period.

## DISCUSSION

Our study is the first to examine the viability of a pre-recorded and remote learning program for EM content in Rwanda. Our results demonstrate that EM residents in Rwanda were receptive to pre-recorded and remote learning as a supplemental tool to their current curriculum. Our qualitative and quantitative data illustrate the relevance and perceived effectiveness of the pre-recorded lectures. Moreover, participants found most of the lectures to be interesting and enjoyable. Notably, participants valued the accessibility of the pre-recorded modality, identifying it as a cost-effective and convenient way to learn new perspectives from speakers external to their institution. In terms of related studies, a study was conducted in remote teaching of Anesthesia that demonstrated effective learning outcomes via pretest-posttest design. This study did not address learner satisfaction or technical challenges, however. ^29^

Although the pre-recorded method was well-received among Rwandan residents, some still preferred in-person lectures, especially when the content of the lecture was considered difficult. Given that some learners perform better in the traditional and familiar model of in-person lectures, this result was to be expected. An obstacle in the program’s implementation was the navigation of network issues and quality of sound on the delivery-side, a technical difficulty that must be considered and addressed in future iterations of program implementation. A distance learning/remote lecture study was conducted in Uganda, centered around obstetrics and gynecology content, with similar findings regarding technological challenges. ^30^

Participants provided positive and negative comments, as well as constructive suggestions on how to improve the pilot program. Many suggested that the lectures should be supplemented with additional materials or resources, such as online or paper handouts. Another suggestion was to coordinate opportunities to apply the concepts presented in the lectures clinically for additional, hands-on practice. Such a suggestion was unsurprising, given that the majority of collected responses (~92%) were from self-reported first- and second-year residents. Lastly, participants requested subtitled videos and access to the recorded lectures even after the live presentation session for further review. It is likely that the implementation of such direct, practical, and actionable requests will bolster the strength of the pre-recorded teaching program for Rwandan EM residents.

### Limitations

As with any study, this project has limitations. Our sample size includes an average of 11 residents per teaching session. This was a census sample within one center; a larger study will be needed to determine generalizability. The questions utilized for the survey were not corroborated by a validated scale. It is possible that the respondents did not understand the questions in the same way as the writers, especially across differing cultural settings. Furthermore, the topics chosen for lectures were identified by the Rwandan faculty, but we did not ask residents for their recommendations. They may have identified other topics to cover based on their perceptions of educational needs. Mixing students of varying background education influences how lectures are delivered as well as students’ ability to grasp concepts. Some students with less training may get intimidated by those with higher training, thus affecting uptake, and our learners were mixed across post-graduate level. Some learners may have experienced lecture fatigue, which is a known limitation of lecture- and video-based learning. Finally, our purpose was to assess acceptance of a pre-recorded learning initiative and satisfaction with the process due to immediate needs. Thus, we did not assess knowledge retention, practice change, or clinical outcomes.

## CONCLUSION

The viability of remote or pre-recorded teaching as a supplemental teaching method for EM curricula in a resource-limited setting has large implications. The COVID-19 pandemic has shed light on many types of disparities – including educational – on domestic and international levels. As the world becomes increasingly interconnected, novel teaching approaches can and should be leveraged to reduce such disparities, especially in places where EM remains a nascent specialty. Pre-recorded teaching offers a cost-effective avenue that can simultaneously use crowdsourcing to create and disseminate much-needed medical knowledge–hopefully translating into reduced morbidity and mortality rates for many rural or resource-limited settings. Further studies are needed to assess knowledge retention, practice change, and clinical outcomes.
